# Developing a Questionnaire on Knowledge, Perceptions and Application of Vascular-Aging Measurements

**DOI:** 10.3390/jcdd10020080

**Published:** 2023-02-14

**Authors:** Areti Triantafyllou, Stavria-Artemis Elia, Chloe Park, Rachel E Climie, Christopher C. Mayer, Ioana Mozos, Giacomo Pucci, Thomas Weber, Andrie G. Panayiotou

**Affiliations:** 1 3rd Clinic of Internal Medicine, Papageorgiou Hospital, Aristotle University of Thessaloniki, 56403 Thessaloniki, Greece; 2 Cyprus International Institute for Environmental and Public Health, Cyprus University of Technology, 3041 Limassol, Cyprus; 3 MRC Unit for Lifelong Health and Aging, UCL, London WC1E 6BT, UK; 4 Menzies Institute for Medical Research, University of Tasmania, Hobart 7000, Australia; 5 Medical Signal Analysis, Center for Health & Bioresources, AIT Austrian Institute of Technology GmbH, 1210 Vienna, Austria; 6 Department of Functional Sciences-Pathophysiology, Center for Translational Research and Systems Medicine, “Victor Babes” University of Medicine and Pharmacy, 300041 Timisoara, Romania; 7 Department of Medicine and Surgery, University of Perugia, Unit of Internal Medicine, Terni University Hospital, 05100 Terni, Italy; 8 Cardiology Department, Klinikum Wels-Grieskirchen, 4600 Wels, Austria

**Keywords:** vascular aging, questionnaire development, PWV

## Abstract

Background: Vascular age (VA) is independent and chronological age for assessing cardiovascular disease (CVD) risk. However, tools for the implementation of VA are currently lacking. We aimed to develop a questionnaire to assess the current knowledge gaps related to VA and barriers to its implementation in routine practice. Methods: Using a stepwise mixed-method approach, a quantitative questionnaire was constructed in four phases: (1) basic item generation and the development of a semi-qualitative questionnaire (SQQ); (2) dissemination to the VascAgeNet extended network and an analysis of the semi-qualitative questionnaire responses; (3) the development of a quantitative questionnaire (QQ); and (4) an assessment of the content and face validity and internal reliability in an additional sample. Results: Based on six main topics initially identified through an expert panel, a SQQ was developed and disseminated. Finally, a 22-item QQ was developed, with questions grouped around three main themes: knowledge of VA and its risk factors; perceptions and beliefs regarding the importance and contribution of VA to risk classification; and the application of VA measurements in clinical and research practice and its potential limitations (Cronbach’s alpha between 0.920 and 0.982 for all three categories). Conclusion: We report the development of a QQ on VA addressed to both clinicians and non-clinicians aiming to assess their knowledge, perceptions and application of VA measurements.

## 1. Introduction

Cardiovascular disease (CVD) remains the leading cause of morbidity and mortality globally, claiming over 17 million deaths every year according to the World Health Organization [1]. Many different scores, such as the Framingham risk score and the European Systemic Coronary Risk Evaluation [2], have been developed to predict CVD risk and to identify individuals at high cardiovascular risk. However, these scores, using traditional risk factors, such as age, sex, smoking, blood pressure and cholesterol levels, misclassify approximately 40% of patients, leading to undertreatment or later diagnosis [3,4]. In order to reduce this high percentage of misclassification, research in recent years has focused on identifying other tools to improve cardiovascular-risk prediction. The identification of vascular aging (VA), as a tool for identifying asymptomatic CVD risk, has been discussed thoroughly in recent years [5,6]. Data from large meta-analyses and cohort studies provide evidence that VA, especially as assessed by pulse-wave velocity (PWV), can serve as a strong predictor of future cardiovascular (CV) events and all-cause mortality, independently of chronological age [7,8]. Despite recent technological developments in the assessment of VA, including a variety of non-invasive techniques, such as PWV, augmentation index, pulse-contour analysis, etc., unmet needs and challenges still exist, limiting its assessment and use in routine clinical practice.

Therefore, VascAgeNet (Network for Research in Vascular Aging, www.vascagenet.eu, accessed on 1 January 2023), is working to refine, harmonize and promote the use of vascular aging biomarkers in order to improve clinical practice and reduce the burden of CVD globally [9]. To effectively increase awareness and education related to VA and to better promote VA to healthcare professionals (action aim), it is essential to first identify any gaps in knowledge and potential obstacles in the implementation of VA assessment in both clinical practice and research settings. Although health providers’ perspectives on other CV risk factors, such as hypertension and diabetes, have been well studied, there are currently, to the best of our knowledge, no studies evaluating the knowledge and perceptions, nor of the implementation, of VA measurements [10,11,12].

Therefore, the present study aimed to develop a tool that could be used by health providers and scientists alike to assess the knowledge gaps related to VA and barriers to its implementation in routine practice in both research and clinical settings for various disciplines.

## 2. Materials and Methods

**Figure 1:**
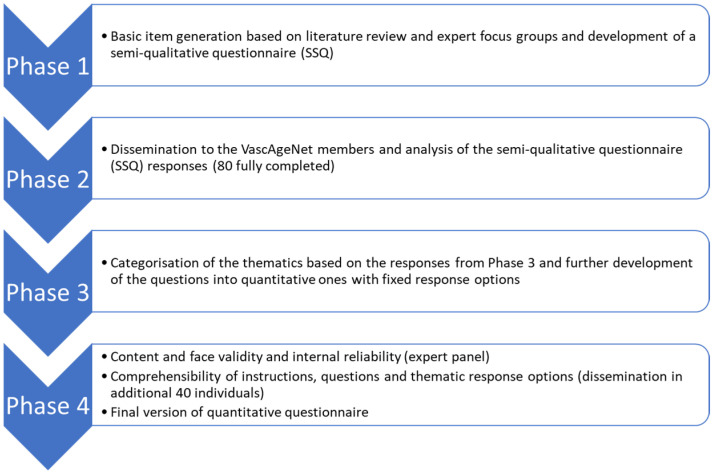
Flow chart of the four development phases of the questionnaire.

Through a stepwise mixed-method approach, the Vascular Aging Perceptions and Implementation Questionnaire (VAPIQ) was constructed in 4 phases: (1) basic item generation and development of a semi-qualitative questionnaire; (2) dissemination and analysis of the semi-qualitative questionnaire responses; (3) development of the quantitative questionnaire; (4) assessment of content and face validity and internal reliability, as well as comprehensibility of the instructions, questions and thematic response options and time to complete (Figure 1).

Phase 1: Basic item generation and development of a semi-qualitative questionnaire

After a literature review, an expert focus group (VascAgeNet Working Group, 6 members) determined six main questions/topics to be addressed, based on the VascAgeNet Action goals (Table 1). These six topics were further developed by an additional expert panel (Working Group 5, WG5) into a semi-qualitative questionnaire, in order to allow an exploratory approach into knowledge and perceptions [13]. Questions were grouped around three main themes, i.e., knowledge of VA and its risk factors, perceptions and beliefs regarding the importance and contribution of VA to risk classification and application of VA measurements in clinical and research practice and potential limitations. The questionnaire was anonymous, but it included a section on basic demographic and occupational information. Questions regarding professional background and practice (e.g., computer scientist, general practitioner, cardiologist, etc.) were left open given the wide range of disciplines involved in VA and in VascAgeNet. Question items were reviewed and agreed upon by the WG5 expert panel and the VascAgeNet CORE group for additional information, visualization of the questions and grammar issues. Study data were collected and managed using REDCap electronic data-capture tools hosted at the Cyprus University of Technology.

Phase 2: Dissemination and analysis of the semi-qualitative questionnaire (SSQ) responses

The SSQ questionnaire was made available online and a survey invitation was sent out to all VascAgeNet participants via email and additionally included in the newsletter for August 2020. People who had participated in the first phase (expert working groups) were excluded from completing the questionnaire. Participants were asked to answer the questionnaire to the best of their knowledge, without reviewing any additional material.

Responses to the open-ended questions were analyzed using the Directed Approach to Qualitative Content Analysis [14]. The goal of the directed approach is to validate or extend conceptually a theoretical framework. Each transcript was read through several times by 3 independent researchers and the codes/themes were extracted for each question based on the answers obtained and agreed after discussion. Finally, subgroups were created to minimize the number of answers to the quantitative questionnaire in the next phase of development.

Phase 3: Development of the quantitative questionnaire

Following the identification of the thematic groups for all responses, open-ended questions were transformed into closed-ended questions by initially categorizing the response options into larger groups and then providing additional options as needed, in order to include all relevant content from the qualitative analysis of the responses. Questions were additionally organized around topic areas and a few additional questions on participation in professional societies were added to better understand the profiles of responders and dissemination channels for future surveys. The final quantitative questionnaire consisted of 22 questions (items), some of which also included additional drop-down questions based on the respondents’ answers. The REDCap electronic data-capture tool hosted at the Cyprus University of Technology was again used for study-data collection and management.

Phase 4: Content and face validity and internal reliability

The resulting quantitative questionnaire was further tested and refined by initially assessing content validity on the final set of questions using the panel of experts from VascAgeNet (Working Group 5 and Core group). The expert panel assessed content validity by examining whether the items were representative of the content they intended to measure, in addition to their relevance and appropriateness for the intended research questions. The experts also commented on how the questions were transformed into closed-ended questions and whether any response option should be added or removed. Furthermore, the final version of the questionnaire was completed by an additional sample of 40 healthcare professionals outside of the VascAgeNet, who had not seen any previous versions, to further assess face validity, as well as comprehensibility, of the instructions, questions and thematic-response options and time to complete. Given that this is questionnaire was mainly intended to explore the knowledge base and potential gaps in knowledge around VA and knowledge that can be fragmented [15], we chose not to pursue principal-component analysis methods in this context so as not to lose any potential information and allow for some potential overlap in the items included.

Internal reliability was tested using Cronbach’s alpha, which indicates whether items measure the same concept/construct [16]. Given that questions were grouped around three main themes, i.e., knowledge of VA and its risk factors, perceptions and beliefs regarding the importance and contribution of VA to risk classification, application of VA measurements in clinical and research practice and potential limitations, we tested whether items in these three categories correlated well with each other, in addition to the internal consistency of all items included in the questionnaire.

## 3. Results

Phase 1: Basic item generation and development of the semi-qualitative questionnaire

Table 1 depicts the initial six main questions/topics identified. The semi-qualitative questionnaire was constructed around these six main topics. Special emphasis was placed on the implementation (and potential limitations) of VA measurements in everyday clinical and research practice because of the lack of recorded data in this field.

**Figure 2:**
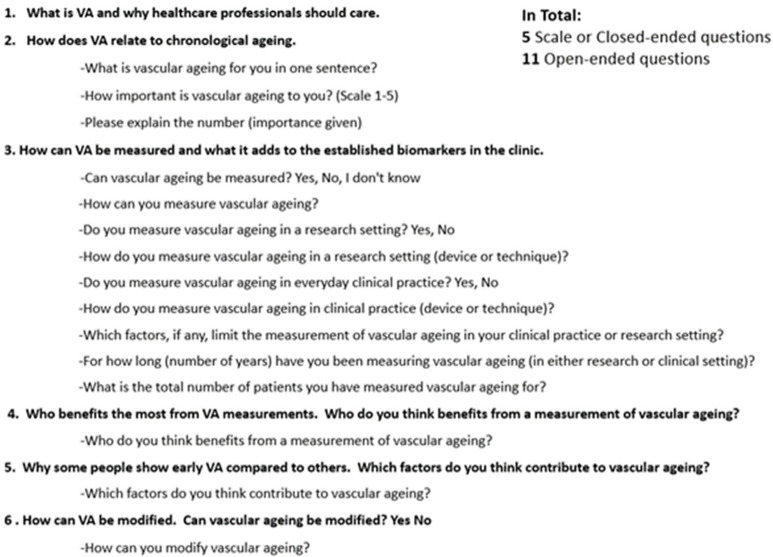
Development of the SSQ from the six main topics.

The expansion of the six initial questions to the semi-qualitative questionnaire used in Phase 2 is shown in Figure 2.

Phase 2: Dissemination and analysis of the semi-qualitative-questionnaire responses

A total of 105 participants, aged 42.6 ± 10.5 years (60.6% male) completed the semi-qualitative questionnaire. Out of the 105 responders, 21 completed only the demographics section. Only four completed one of the open-ended questions and were thus excluded from the analysis. The baseline characteristics of the included and excluded participants, according to the criteria described above, can be found in Table 2. In total, 80 completed questionnaires were used in the qualitative analysis for the creation of the final quantitative questionnaire.

### 3.1. Knowledge of VA (Measurements) and Its Risk Factors

We started by simply asking whether VA can be measured and, if so, how. The vast majority of the participants (93.7%) replied positively (VA can be measured) and 6.3% stated that they did not know or left their response blank. Regarding the question on “How they measure VA”, there was a plurality of answers stating both methods and specific devices, which were then grouped into respective categories (for example, PWV measurement, intima-media thickness (IMT) and carotid stiffness were all included in the category “ultrasound imaging” and flow-mediated dilation (FMD) was included in the category “endothelial function markers”). Of those who answered that VA can be measured, 28.2% provided a general description or did not answer at all. The risk factors contributing to VA were grouped into the following three categories: genetics (41%), lifestyle factors (34%) and other CVD factors (25%). The categories of “Lifestyle” and “Other CVD risk factors” were further expanded to include the following response options: physical activity, diet/nutrition, smoking, stress, blood pressure, diabetes mellitus, lipids and others.

When asked whether VA can be modified and how, the vast majority responded positively (97.5%) and the responses were grouped into the following categories: diet, physical exercise, smoking, stress, inflammation, oxidative stress, reduction in glucose and BP and drug-treatment administration, including antihypertensive, lipid-lowering drugs, anti-TNF and glucose-lowering drugs.

**Figure 3:**
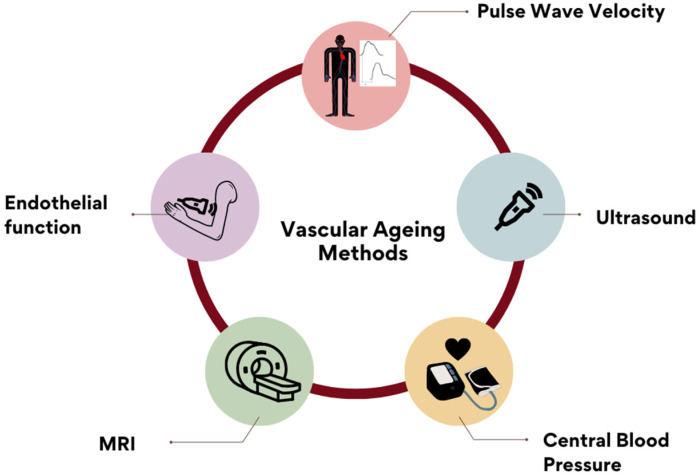
Reported methods for measuring AS included: PWV (48.7%), ultrasound imaging, central blood pressure, endothelial-function markers and magnetic-resonance imaging. Other answers included: coronary-calcium score, blood-pressure measurement, catheterization methods, oscillometry, photoplethysmography, augmentation index and biochemical markers. These were recorded and included in the ‘Others’ open category.

The most popular reported methods for assessing arterial stiffness are shown in Figure 3.

### 3.2. Perceptions and Beliefs Regarding the Importance and Contribution of VA to Risk Classification

When asked to assign a grade of (personal) importance to VA, 88.6% of the participants responded that VA is important or very important, 7.6% stated that it is neither, 2.5% claimed it is not important and 1.3% reported that they did not know. Those assigning the highest grade did so mostly due to personal research interests. Other reasons included the belief that VA can improve primary preventions and treatment strategies, act as a marker of cardiovascular and other diseases and provide an estimation of overall health. The reasons for a low grade of significance included the belief that VA does not add to the existing risk markers, the argument that it is not yet a validated marker and the suggestion that it cannot improve treatment strategies. All the above were included as response options in the resulting closed question, in addition to the following, based on the expert panel review: “VA improves cardiovascular risk evaluation, lack of specific guidelines, it is/it is not a useful marker in routine clinical practice.”

Regarding the question, “What does vascular aging add to the established biomarkers in the clinic?” the responses were mixed, with 50.6% stating that VA is beneficial, 26.8% not responding, 8.8% stating that it adds little, 5.1% stating that they did not know and 10.1% giving a response that was not deemed relevant to the question. The specific responses included the following: VA offers a comprehensive overview of the status of the vascular system; it acts as a primordial CVD indicator, leading to early, better, reclassified, independent and individualized CVD prediction; and it can be used as a visual motivational tool for patients and as a treatment guide for individualized care.

When asked to identify who benefits from the measurement of vascular aging, 45% mentioned multiple groups, while 55% specified only one. Examples of the answers included: patients, doctors, researchers, society, healthy people, patients with hypertension, diabetes mellitus and obesity. Multiple answers were also recorded that identified patients at various cardiovascular-risk levels who would benefit more from the estimation of VA- and, therefore, CV-risk categories (low, medium, high); these were included as additional response options in the final quantitative questionnaire. Finally, different age groups were also mentioned (children and adolescents; healthy people aged 18–40; healthy people aged 40–65; healthy people aged >65) and included as response options.

### 3.3. Application of VA Measurements in Clinical and Research Practice and Potential Limitations

**Figure 4:**
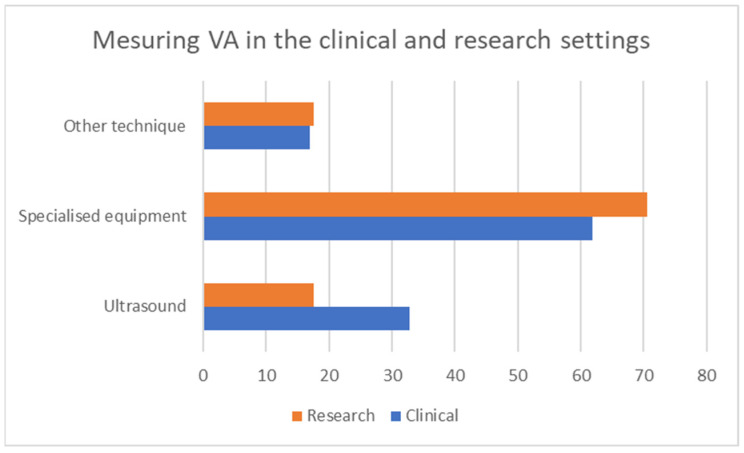
Categories of responses on measuring VA by setting.

Twenty-four percent of the participants stated that they currently measure VA in their clinical practice, while 67% stated that they do so in their research practice. The grouped responses for the question, “How do you measure vascular aging in clinical or research practice (device or technique)?” are shown in Figure 4. The question was split into two, with one focusing on devices and one on methods in the final quantitative questionnaire, so as to include all the possible responses. The possible responses to the first question included: carotid-femoral-pulse-wave velocity, brachial-ankle-pulse-wave velocity, carotid-intima-media thickness, augmentation index, central blood pressure, carotid stiffness, retinal flicker and endothelial functioning. The responses to the second question included: Sphygmocor, AtCor, Complior, Mobil-O-graph, Pulse Pen, Popmeter, Arteriograph, Art Lab, Endopat, VascAssist, Vascular Explorel and Vicorder.

Regarding the limitations, the predominant answer was time (27.8%), followed by the cost of the equipment, the fact that the test is not included on the approved list of refundable tests and the lack of guidelines (13.6% for each). Other limitations reported were that validated or reliable devices do not exist (12.6%) and that there is no clinical benefit (11.4%). Other, less common answers included the high variability of the measurement, patient compliance, a lack of reference values, special patient conditions (obesity, atrial fibrillation) and the suggestion that no specific guidelines on how VA can modify treatment exist. Only 2.5% stated that there are no limitations and 31.9% stated that they could not respond or did not answer at all.

Phase 3: Development of the quantitative questionnaire

Based on the results of the qualitative analysis and by incorporating the questions about demographic and social characteristics, a 22-item quantitative questionnaire was developed. The questionnaire included four questions on the perceptions/beliefs of the participants regarding VA (Q9, 10, 18, 22), four questions regarding their knowledge on VA (Q11, 12, 13 and 19) and four questions regarding the implementation of VA in clinical and research practice (Q14, 15, 16, 17). The answer options varied from a Likert-type scale to pre-specified categories with several sub-options. All the responses additionally included an “Other” option, allowing the participants to further specify their responses. The final version of the quantitative questionnaire can be downloaded from the link in the Supplementary Materials.

Phase 4: Content and face validity and internal reliability

The content validity was assessed by the expert group who participated in phase 2. The group concluded that the questions and possible responses were both relevant and appropriate. Regarding the face validity, this was further assessed by an additional group of health-related professionals who responded to the final quantitative version of the survey. They also deemed the questions and available response options both relevant and appropriate. Based on their responses, we decided to also keep the additional option, “Other,” with room for further elaboration in all the questions, as we received two responses that we did not include in the pre-specified categories to the questions regarding “occupation,” “specialty,” and “conditions that might affect VA.” For the internal reliability, as mentioned in the Methods section, we categorized the questions into three main themes: (a) knowledge of VA and its risk factors; (b) perceptions and beliefs around the importance and contribution of VA to risk classification; and (c) the application of VA measurements in clinical and research practice and its potential limitations. After removing the initial demographic questions, four questions, with their respective pre-specified responses (41 response items), were included in the first category, four (34 response items) in the second and four (43 response items) in the third. Cronbach’s alpha was estimated for each category, as shown in Table 3. As can be seen, the reliability coefficient (alpha) for all three categories was very high, indicating a high level of internal consistency among the items grouped in the same category. When further examining all the non-demographic questions included (118 response items) in an attempt to assess whether the entire questionnaire was consistent in capturing information around the same concept (i.e., vascular aging), the Chronbach’s alpha was again high (0.9649), indicating a very level of high consistency across all the items included in the questionnaire.

## 4. Discussion

There is accruing evidence that vascular-aging measures can help improve cardiovascular-risk prediction [17,18,19] and detect those at high risk earlier [6,17,18,19]. However, despite increasing knowledge about its value and the technological advances in its measurement, its implementation in clinical practice is lagging. To address this and to effectively increase awareness and education related to VA for healthcare professionals, it is essential to first identify any gaps in knowledge and potential obstacles in the implementation of VA assessment in both clinical practice and research settings. To this end, the VascAgeNet group developed a tool to evaluate awareness and perceptions around VA, as well as the implementation of VA measurements in the everyday practice of healthcare providers and researchers, using a mixed-method approach. This allowed us to capture information without making any prior assumptions that could have introduced bias into the developed quantitative questionnaire. As one of the main aims of the questionnaire was to assess the knowledge base around VA and the knowledge around a topic may be fragmented [15], the development of the questionnaire was not underpinned by a theoretical framework. Repeated evaluation (content layout, wording, the ordering and addition of questions and editing) was also implemented throughout the process, while a content analysis of the responses was performed by three independent researchers. The internal consistency of the questionnaire (as assessed by Cronbach’s alpha) was found to be very satisfactory (between 0.920 to 0.982 for all the categories) based on the grouping of the questions around the three main categories. This indicated that the questions included in the final version of the questionnaire did indeed capture the three elements/themes that we set out to capture, namely the knowledge base around VA and its risk factors, the perceptions and beliefs around the importance and usefulness of VA and, finally, its application in both research and clinical settings.

The strengths of this study include (1) the stepwise development and the qualitative recording and thematic analysis and (2) participant selection. The final response options were based on the items and thematic topics originating from the recording and the qualitative analysis of a varied sample of participants from multiple scientific backgrounds, assuming no specific theoretical framework. This allowed us to avoid any preconceptions by employing exploratory, qualitative research, which helps provide unbiased respondent thoughts or perceptions, without restricting the questions to individual experts’ opinions. Based on this work, we now propose a quantitative questionnaire, which can be used in larger surveys without the complex recording, tabulation and analysis required by qualitative approaches [20,21].

The participants came from the extended VasAgeNet network, which includes both researchers and clinicians and, thus, represented members of the population of interest, ensuring that the questionnaire reflected their perspectives and that the items were acceptable, comprehensive and relevant to their condition [22]. Furthermore, the participants had various degrees of experience, came from several different countries/healthcare systems and had various scientific backgrounds. This was particularly important as a number of different scientific disciplines and professional specialties are currently working on or are interested in VA and, therefore, the identification of s specific issues/gaps that are potentially relevant to different specialties may be pivotal in the use of targeted approaches to increase knowledge and awareness, compared with general approaches. As cardiovascular diseases are also leading causes of death in patients with other diseases, such as renal [23] and oncology patients [24,25], assessing the relevant knowledge base on VA and the potential limitations of its use could be useful in various clinical disciplines, as they could also benefit from the implementation of VA measurements for cardiovascular-risk assessment in their patients. Allowing for some redundancy in certain response options was deemed appropriate in order to cover all the possible options selected by responders from different disciplines, who might not have been represented in the development of the tool. The results from a larger cohort that responded to the final quantitative questionnaire are pending; they will further inform our efforts.

The limitations of this study include the lack of test–retest data for the final version, the subjectivity related to the use of questionnaires and its cross-sectional nature. However, it should be mentioned that test–retest reliability is desirable mostly in measures of constructs that are not expected to change over time, such as personality traits [26]. It is possible that the participants in the survey were motivated to research some of the concepts after filling in the survey and, thus, the test–retest may have been biased.

## 5. Conclusions

In conclusion, the present study aimed to develop and pilot a survey questionnaire for people working in or with an interest in the field of VA cardiovascular disease, in order to capture information on the current knowledge, perceptions and application of VA. The questionnaire was developed following a literature review to identify relevant content, in addition to some exploratory work and discussions with panels of experts in the field. It can be now used more extensively in large surveys targeting various clinical and non-clinical specialists, such as cardiologists, primary-care nurses, dieticians, exercise physiologists, etc., who could potentially benefit from its use. In addition, the findings of this study can help to guide the design of targeted and effective educational, awareness, prevention and treatment strategies.

## Figures and Tables

**Table 1:** The six main questions/topics around VA originally identified by the expert focus group.

1. What is VA and why should healthcare professionals care?
2. How does VA relate to chronological aging?
3. How can VA be measured and what does it add to the established biomarkers in the clinic?
4. Who benefits the most from VA measurements?
5. Why do some people show early VA compared to others?
6. How can VA be modified?

**Table 2:** Demographics of participants included in and excluded from the qualitative analysis (phase 2).

	Final Participants (*n* = 80)	Excluded Participants (*n* = 25)	*p*
Age (years)	42.6 ± 10.5	42.0 ± 9.0	0.759
Sex (Male) %	60.6	53.8	0.508
Clinicians/Other %	44.9/55.1	38.5/61.5	0.651

**Table 3:** Reliability coefficient (Cronbach’s alpha) for the final questionnaire.

Thematic Categories	N of Response Items on Scale	Cronbach’s Alpha
(a) Knowledge of vascular aging (VA)	41	0.9816
(b) Perceptions and beliefs around VA	34	0.9206
(c) Application of VA measurements	42	0.9316
Overall (all response items)	118	0.9649

## Data Availability

Data are available upon request.

## Supplementary Materials

The following supporting information can be downloaded at: https://www.mdpi.com/article/10.3390/jcdd10020080/s1: Final version of Quantitative Questionnaire.
