# Supplementary material for: Developing a Questionnaire on Knowledge, Perceptions and Application of Vascular-Aging Measurements
Source: J Cardiovasc Dev Dis. 2023 Feb 14;10(2):80. doi: 10.3390/jcdd10020080 (PMC9965266; doi:10.3390/jcdd10020080)
Supplement: Supplementary file 1 [file jcdd-10-00080-s001.zip › jcdd-2141021-supplementary.pdf]

Knowledge and Perceptions about Vascular Ageing

VascAgeNet Survey on Vascular Ageing

Participant Information Sheet

The following is a short survey conducted by the COST Action VascAgeNet (CA18216). COST Action CA18216 is supported by COST (European Cooperation in Science and Technology, http://www.cost.eu. This survey was created by the VascAgeNet Public Engagement team. Principal Investigators: Dr Andrie Panayiotou (Cyprus International Institute for Environmental and Public Health, andrie.panayiotou@cut.ac.cy), Dr Areti Triantafyllou (University of Thessaloniki, artriant@auth.gr) and Dr Chloe Park (University College London, chloe.park@ucl.ac.uk); and the researcher team Dr Rachel Climie, (Baker Heart and Diabetes Institute, Rachel.Climie@baker.edu.au), and Dr Christopher Mayer (AIT Austrian Institute of Technology, Christopher.Mayer@ait.ac.at).

Please take your time to read the following information carefully.

Why is the study being conducted?The purpose of this survey is to investigate knowledge and perceptions about vascular ageing.

What does participation involve?We value the opinions of all individuals interested in the cardiovascular disease field. If you agree to participate, you will be asked to answer questions on your knowledge, perceptions and experience in measuring vascular ageing.

The survey will take approximately 5-8 minutes to complete.

Do I have to take part?Your participation in this research project is entirely voluntary. If you do agree to participate you can withdraw from the survey without comment or penalty. You may stop at any time by closing your browser.

However, as the survey does not request any personal identifying information, once it has been submitted, it will not be possible to withdraw.

What will happen to the data that is being collected?Data are anonymous and will be archived at the Cyprus University of Technology and University College London. This survey has been approved by the Cyprus National Committee on Bioethics (EEBK 2020.01.103) and University College London (17999/001). The data will be used solely for research purposes and will inform the research teams' outputs including, publications. Your data will be processed so long as it is required for the duration of the 4 year COST Action.

What are the possible benefits for me if I take part?It is expected that this research project will not directly benefit you. The outcomes of the research, however, may benefit the development of resources and training for clinicians and researchers as well as policy and practice reform.

What are the possible risks for me if I take part?There are no risks associated with your participation in this survey.

Are there any ethical considerations?This study has been reviewed and approved by the UCL Research Ethics Committee and the Cyprus National Committee on Bioethics. If you have any concerns or comments regarding the way this research has been conducted, please contact Dr Chloe Park (chloe.park@ucl.ac.uk). If your concerns have not been satisfactorily addressed by Dr Park, please contact the Chair of the UCL Research Ethics Committee (ethics@ucl.ac.uk).

Who can I contact if I have any questions or require more information about the project?Please contact the VascAgeNet Public engagement team. Project's Principal Investigators: Dr Andrie Panayiotou (andrie.panayiotou@cut.ac.cy), Dr Areti Triantafyllou (artriant@auth.gr) and Dr. Chloe Park (chloe.park@ucl.ac.uk). Researcher Team: Rachel.Climie@baker.edu.au and Christopher.Mayer@ait.ac.at. For more information on

VascAgeNet please visit www.vascagenet.eu/

How do I give my consent to participate?Consent will be assumed through completion of the survey. By clicking next, you confirm that you have read and understood the information provided above and agree to participate anonymously.

Please answer all questions to the best of your current knowledge and without reviewing additional resources.
By clicking next, you confirm that you have read and understood the information provided above and agree to participate anonymously.

For more information on VascAgeNet please visit www.vascagenet.eu/

---

1 Are you a member of any of the following professional societies? (Please select all that apply)

☐ No
☐ European Society of Hypertension
☐ International Society of Hypertension
☐ Artery Society
☐ North American Artery Society
☐ European Society of Cardiology
☐ British and Irish Hypertension Society
☐ High Blood Pressure Research Council of Australia
☐ Australian Cardiovascular Alliance
☐ Other

If Other, please specify

____

---

2 How did you hear about this survey? (Please select all that apply)

☐ Facebook or other social media
☐ Professional society (email/newsletter)
☐ Friend/colleague
☐ Other

2a If Other, please specify

____

---

3 Age (in years)

____

---

4 Gender

◯ Male ◯ Female ◯ Diverse ◯ Prefer not to say

5a Country of current residence

____

5b City of current residence

____

6 Occupation (Please select all that apply)

☐ Student
☐ Graduate student
☐ PhD student
☐ Researcher (academic/research Institution)
☐ Academic
☐ Clinical Doctor
☐ Other

6a Resident or Specialist ☐ Resident ☐ Specialist ☐ Other

6b If Other, please specify

____

7 Please specify your specialty

☐ Clinical specialty
☐ Biomedical engineer/computer scientist
☐ Physiologist
☐ Epidemiologist/statistician/mathematician
☐ Basic science researcher
☐ Other

7a Please specify your clinical specialty

☐ Cardiology
☐ Internal Medicine
☐ General Practice
☐ Other

7b If Other, please specify

____

8 Place of work

☐ Clinic in a university hospital
☐ Public hospital
☐ Private hospital
☐ Public health center
☐ Private practice
☐ Academic/research institution
☐ Industry
☐ Other

8a If Other, please specify

____

This section will consist of questions on vascular ageing. Please provide an answer to the best of your knowledge

		1	2	3	4	5	I don't know
9	How important is vascular ageing to you? With 1 being least important, and 5 very important	○	○	○	○	○	○

9a Please explain the number (importance given) (Please select all that apply)

- ☐ Personal (research) interest
- ☐ Improves primary prevention
- ☐ Improves cardiovascular risk evaluation
- ☐ It does not add to the existing risk markers
- ☐ It improves treatment strategies
- ☐ It is a marker for the overall effect of several risk factors
- ☐ It is not a validated marker
- ☐ It is a marker of overall health
- ☐ It is an underlying cause of various diseases
- ☐ There are no specific clinical guidelines
- ☐ It is a useful marker in clinical practice
- ☐ It does not improve treatment strategies
- ☐ It is not a useful marker in clinical practice
- ☐ Other

10 Who do you think benefits from a measurement of vascular ageing? (Please select all that apply)

- ☐ No one
- ☐ Everyone
- ☐ Children and adolescents
- ☐ Healthy people aged 18-40
- ☐ Healthy people aged 40-65
- ☐ Healthy people aged >65
- ☐ People with positive family history of cardiovascular disease
- ☐ People with other non-cardiovascular comorbidities
- ☐ People at low cardiovascular risk
- ☐ People at intermediate cardiovascular risk
- ☐ People at high cardiovascular risk
- ☐ People with obesity
- ☐ People with hypertension
- ☐ People with diabetes mellitus
- ☐ Other

10b If Other, please specify

____

11 Which factors do you think contribute to early (i.e. premature) vascular ageing? (Please select all that apply)

- ☐ Age
- ☐ Sex
- ☐ Lifestyle factors
- ☐ Other cardiovascular risk factors
- ☐ Genetics/family history
- ☐ Environmental factors
- ☐ Inflammation/oxidative stress
- ☐ Other

11a If lifestyle factors, please specify

☐ Diet/Nutrition
☐ Physical activity
☐ Smoking
☐ Stress

11b If Other cardiovascular risk factors, please specify

☐ Hypertension
☐ Diabetes mellitus
☐ Hyperlipidemia
☐ Obesity
☐ Kidney dysfunction

11c If Other, please specify

____

12 Can vascular ageing be modified?

◯ Yes ◯ No

12a How can vascular ageing be modified? (Please select all that apply)

☐ Diet/nutrition
☐ Physical activity
☐ Antihypertensive medication
☐ Anti-lipid medication
☐ Glucose lowering medication
☐ Anti-TNF medication
☐ Stress
☐ Quitting/reducing smoking
☐ Other

12a If Other, please specify

____

13 Can vascular ageing be measured?

◯ Yes ◯ No ◯ I don't know

13a How can vascular ageing be measured? (Please select all that apply)

☐ Ultrasound Imaging
☐ Pulse wave velocity measurements
☐ Central blood pressure
☐ Endothelial function markers
☐ Magnetic Resonance Imaging
☐ Other

14 Do you measure vascular ageing in a research setting?

◯ Yes ◯ No

14a How do you measure vascular ageing in a research setting ? (Please select all that apply)

☐ Ultrasound imaging
☐ Specialized equipment
☐ Other

14b If Other, please specify

____

14c Please specify equipment used

☐ Sphygmocor
☐ AtCor
☐ Complior
☐ Mobil-O-Graph
☐ Pulse pen
☐ Popmeter
☐ Arteriograph
☐ Art Lab
☐ Endopat
☐ VascAssist
☐ Vascular Explorer
☐ VICORDER
☐ Other

14d Please define measurements taken (Please select all that apply)

☐ Carotid-femoral pulse wave velocity
☐ Branchial-ankle pulse wave velocity
☐ Carotid intima media thickness
☐ Augmentation index
☐ Central blood pressure
☐ Carotid stiffness
☐ Retinal flicker
☐ Endothelial function
☐ Other

15 Which factors, if any, limit the measurement of vascular ageing in your research setting? (Please select all that apply)

☐ Time
☐ Cost of equipment
☐ Not included in approved list of refundable tests
☐ Lack of guidelines
☐ Lack of operator expertise
☐ Not a validated marker
☐ No validated device
☐ No clinical benefit
☐ I don't know enough about it
☐ Other
☐ No limitations

16 Do you measure vascular ageing in everyday clinical practice?

◯ Yes ◯ No

16a How do you measure vascular ageing in clinical practice ?

- ☐ Ultrasound imaging
- ☐ Specialized equipment
- ☐ Other

16b Please specify equipment used

- ☐ Sphygmocor
- ☐ AtCor
- ☐ Complior
- ☐ Mobil-O-Graph
- ☐ Pulse pen
- ☐ Popmeter
- ☐ Arteriograph
- ☐ Art Lab
- ☐ Endopat
- ☐ VascAssist
- ☐ Vascular Explorer
- ☐ VICORDER
- ☐ Other

16c Please specify measurements taken (Please select all that apply)

- ☐ Carotid-femoral pulse wave velocity
- ☐ Branchial-ankle pulse wave velocity
- ☐ Carotid intima media thickness
- ☐ Augmentation index
- ☐ Central blood pressure
- ☐ Carotid stiffness
- ☐ Retinal flicker
- ☐ Endothelial function
- ☐ Other

17 Which factors, if any, limit the measurement of vascular ageing in your clinical practice? (Please select all that apply)

- ☐ Time
- ☐ Cost of equipment
- ☐ Not included in approved list of refundable tests
- ☐ Lack of guidelines
- ☐ Lack of operator expertise
- ☐ Not a validated marker
- ☐ No validated device
- ☐ No clinical benefit
- ☐ I don't know enough about it
- ☐ Other
- ☐ No limitations

18 What does vascular ageing add to the established biomarkers in clinic? (Please select all that apply)

☐ A comprehensive overview/lifetime result of the status of the vascular system
☐ A visual motivational tool for patients
☐ A primordial risk factor showing effect before other risk factors
☐ A direct visualization of accumulated effect of other risk factors
☐ A reclassification/identification of patients at risk
☐ An independent/better risk factor for cardiovascular disease
☐ An individualized and more precise picture of risk
☐ A functional marker Vs structural markers
☐ A treatment marker/individualized treatment plans
☐ A risk modifier
☐ Not much/already too many markers in clinic
☐ We need a uniform global approach before clinical use
☐ I don't know

19 Why do you think some people display early (i.e premature) vascular ageing compared to others? (Please select all that apply)

☐ Genetics/(Family) predisposition
☐ Lifestyle choices
☐ Other cardiovascular risk factors

19a If lifestyle choices was selected, please specify

☐ Physical activity
☐ Diet/Nutrition
☐ Smoking
☐ Stress

19b If Other cardiovascular risk factors was chosen, please specify

☐ Blood pressure
☐ Diabetes mellitus
☐ Lipids
☐ Other

20 How long (number of years) have you been measuring vascular ageing (in either research or clinical setting)?

____

21 What is the total number of patients you have measured vascular ageing for?

____

22 What does vascular ageing mean to you? (Please select all that apply)

☐ A marker of overall cardiovascular disease risk
☐ Not a very useful marker
☐ The structural deterioration of the vasculature
☐ The functional deterioration of the vasculature
☐ The physiological ageing of the vasculature
☐ A measure of biological ageing
☐ I don't know enough about it
☐ Other

22a If Other, please specify
